# The beneficial effect of Bifidobacterium breve Br03 and Bifidobacterium breve B632 mixture in acute infectious diarrhea in children

**DOI:** 10.1007/s12602-026-10996-x

**Published:** 2026-03-25

**Authors:** Soner Sertan Kara, Kartal Berkay Gürkan, Eda Somuncu, İlknur Çağlar

**Affiliations:** Faculty of Medicine, Department of Pediatric Infectious Diseases, Aydın Adnan Menderes University, Aydın, Türkiye

**Keywords:** *Bifidobacterium breve*, Diarrhea, Probiotics, Children, Rehydration

## Abstract

Introduction. *Bifidobacterium breve* species have demonstrated potential therapeutic effects on acute diarrhea; however, supporting clinical evidence remains limited. This study evaluates the impact of a combination of *Bifidobacterium breve* BR03 and *Bifidobacterium breve* B632 on the clinical outcomes of children with acute diarrhea. Methods. This randomized, prospective study included children aged six months to five years at a tertiary care center. The intervention group received a probiotic formulation containing *B. breve* BR03 and *B. breve* B632 (five drops/day) alongside peroral zinc (15 mg/day) and oral rehydration solution (ORS) for five days. The control group received ORS and peroral zinc for the same duration. Results. Among the 120 enrolled children, 72 (60%) were boys. The median ages were 32.0 months (18.0–45.0) in the study group and 30.0 months (17.0–55.0) in the control group, with no significant differences in baseline characteristics. Stool frequency was significantly lower in the study group at 24–48 h (2.41 ± 1.99 vs. 3.07 ± 1.71, *p* = 0.020), 48–72 h (0.98 ± 1.31 vs. 1.90 ± 1.58, *p* < 0.001), and 72–96 h (0.44 ± 1.18 vs. 1.03 ± 1.35, *p* < 0.001). The proportion of children experiencing diarrhea at these intervals was also significantly lower in the probiotic group. Conclusion. The combination of *B. breve* BR03 and *B. breve* B632 significantly reduces stool frequency and diarrhea duration in children with acute diarrhea, supporting its potential as an adjunct therapy.

## Introduction

Diarrhea remains a significant yet preventable and treatable global health challenge, particularly in young children. Each year, approximately 1.7 billion cases of diarrhea occur in children, leading to nearly 444,000 deaths in those under the age of five worldwide [1]. The condition primarily arises due to disruptions in the integrity of physical and chemical barriers within the gastrointestinal tract, alongside impairments in intestinal ion transport proteins and channels, ultimately resulting in fluid and electrolyte malabsorption [2]. Infectious agents, including Rotavirus, Norovirus, Escherichia coli, Salmonella spp., and Entamoeba histolytica predominantly cause acute diarrhea. While most cases are self-limiting and resolve within a few days, severe cases can lead to life-threatening dehydration, bacteremia, and sepsis. The cornerstone of pediatric diarrhea management involves supportive care, including adequate fluid and electrolyte replacement, continued breastfeeding or age-appropriate nutrition, and, when indicated, antibiotic therapy. Zinc supplementation is also recommended in children aged six months to five years to reduce the duration and severity of symptoms.

The gut microbiome plays a crucial role in maintaining intestinal and systemic health. Disruptions in microbial balance, or dysbiosis, have been implicated in the pathogenesis of gastroenteritis, inflammatory bowel disease, obesity, autism spectrum disorders, and allergic conditions. A deficiency in beneficial bacteria such as *Bifidobacterium* spp., *Bacillus* spp., and *Lactobacillus* spp., which produce lactase and contribute to gut homeostasis, is associated with an increased susceptibility to diarrhea [3]. These probiotic microorganisms exert protective effects by synthesizing antimicrobial compounds, competing with pathogens for nutrients, enhancing intestinal barrier function, and modulating mucosal immune responses [4, 5]. Given their potential, probiotics have emerged as promising adjuncts in managing acute diarrhea. However, due to the gut microbiota’s complexity, specific probiotic strains’ efficacy varies across different clinical contexts. Current guidelines recommend several strains for diarrhea treatment, including *Saccharomyces boulardii*, *Lactobacillus rhamnosus* GG, *L. reuteri* DSM 17,938, and *L. rhamnosus* 19070-2 in combination with *L. reuteri* DSM 12,246 [6].

Among probiotics, *Bifidobacterium* spp. are Gram-positive anaerobic bacteria that constitute a significant portion of the neonatal gut microbiome, although their abundance declines with age. A previous randomized controlled trial demonstrated that daily administration of *Bifidobacterium breve* BR03 and *B. breve* B632 at a concentration of 10⁸ CFU significantly reduced vomiting episodes, improved stool consistency, and decreased defecation frequency [7]. Despite these promising findings, limited data exist regarding the therapeutic potential of this combination in acute diarrhea. This study aims to evaluate the clinical effects of *B. breve* BR03 and *B. breve* B632 in children with acute diarrhea, providing further insight into their role in pediatric gastroenteritis management.

## Methods

### Study Design and Ethical Considerations

This randomized, prospective study was conducted at the Department of Pediatric Infectious Diseases, Aydin Adnan Menderes University Medical Faculty, Türkiye, between November 1, 2023, and March 1, 2024. The protocol for this study received ethical approval from the institutional review board (Approval No: 2023/175, Date: October 11, 2023). Informed consent was obtained from the caregivers of all participants before their enrollment, adhering to the ethical standards set for clinical research.

### Study Population

The study focused on children aged six months to five years who presented with acute diarrhea. Diarrhea was diagnosed based on the passage of three or more loose or watery stools per day, which occurred within the preceding 24 h. Stool consistency was assessed using the Bristol Stool Chart. Only children who met the inclusion criteria and presented with diarrhea within the 24-hour window were enrolled in the study.

### Inclusion and Exclusion Criteria

Children diagnosed with acute diarrhea during the previous 24 h and within the age range of six months to five years were eligible for inclusion in the study. Children were excluded if they had severe dehydration requiring admission to the critical care unit or if they had chronic diseases such as immunodeficiency, inflammatory bowel diseases, or severe malnutrition. Additionally, the study did not include children who had received antibiotics, probiotics, or any other medications affecting gastrointestinal absorption or motility in the previous month.

### Randomization and Study Groups

Participants were randomized based on the order of their presentation. The first participant was assigned to the control group, the second to the intervention group, and so on. The intervention group received a daily dose of five drops of a probiotic formulation containing Bifidobacterium breve BR03 and B. breve B632 (Symbiosys Bifidrop^®^, Biocodex, France), in addition to peroral zinc (15 mg/day) and oral rehydration solution (ORS) for five consecutive days. In contrast, the control group received only ORS and peroral zinc for the same duration.

### Outcome Measures

The study’s primary outcome was to assess the effect of the combination of B. breve BR03 and B. breve B632 on the frequency of diarrhea episodes over the five-day intervention period. The secondary outcome was the number of children who continued to experience diarrhea at the end of the intervention period (120 h post-treatment initiation). Diarrhea was defined as the passage of loose or watery stools three or more times per day. The caregivers assessed Stool consistency using the Bristol Stool Chart, with a score of less than 5 indicating normal stool consistency.

### Clinical and Anthropometric Assessments

Upon admission, participants were thoroughly assessed regarding their demographic characteristics, including age and gender, and clinical parameters, such as the number and duration of diarrhea and vomiting episodes. Anthropometric measurements, including weight, height, and body mass index (BMI), were also recorded. Additionally, the severity of dehydration was assessed using the Modified Vesikari Score and the Clinical Dehydration Scale (CDS).

Caregivers were contacted daily to report the frequency and consistency of stools, and caregivers used the Bristol Stool Chart for this purpose. Normal stool consistency was classified as a Bristol Stool Chart score below 5. A final clinical examination was performed at the end of the 120-hour treatment period to evaluate the overall outcomes.

### Sample Size Calculation

Due to the absence of a similar study with available data for reference, the sample size calculation was based on the t-test medium effect size. The study aimed for a statistical power of 80%, with a margin of error set at 5%. The study included one hundred twenty participants, with an additional 15–20% buffer for potential dropouts.

### Statistical Analysis

The data were analyzed using SPSS version 17.0 (SPSS Inc., Chicago, IL, USA). The normality of the data was assessed using the Kolmogorov-Smirnov and Shapiro-Wilk tests. Descriptive statistics were computed, including measures of central tendency (mean and median) and dispersion (standard deviation and range). Comparisons between the intervention and control groups were made using the independent **t-test** for normally distributed continuous variables, the Mann-Whitney U test for non-normally distributed continuous variables, and the chi-square test for categorical variables. The risk ratio (RR) with a 95% confidence interval (CI) was used to compare outcomes between the two groups. A p-value of less than 0.05 was considered statistically significant.

## Results

Table 1 shows 150 children with acute gastroenteritis after dropouts as no significant differences were observed in body weight at admission or the end of the episode, height, or clinical scores for dehydration (CDS and Vesikari scores). The duration of diarrhea at admission, number of stools, and number of vomiting episodes in the last 24 h were also comparable between the two groups. Regarding stool results, rotavirus was detected more frequently in the study group (14.3%) compared to the control group (5.6%), while adenovirus and other pathogens were found at similar rates in both groups.

**Table 1:** Demographic and clinical properties of patients at admission

	Study group (n=61)	Control group (n=59)	p
Age (months; median [IQR])	32.0(18.0- 45.0)	30.0(17.0- 55.0)	0.755
Body weight at admission	13.0(10.8- 18.0)	13.0(9.8-16.0)	0.610
Body weight at the end of the episode	13.0(10.8- 18.0)	12.7(9.6-15.9)	0.413
Height	90.7±14.4	90.1±14.5	0.809
CDS score	1.0(0.0-4.0)	2.0(0.0-4.0)	0.703
Vesikari score	10.0(7.0- 11.0)	9.0(7.0-12.0)	0.489
Diarrhea duration at admission	24.0(18.0- 72.0)	40.0(18.0- 72.0)	0.865
Number of stools during the last 24 hours	4.0(3.0-7.0)	4.0(3.0-6.0)	0.907
Number of vomiting during the last 24 hours	2.0(0.0-4.0)	2.0(0.0-3.0)	0.315
Stool results, n(%)			
Rotavirus	8(14.3)	3(5.6)	
Adenovirus	1(1.8)	3(5.6)	
Rotavirus+Adenovirus	0(0)	1(1.9)	
* Salmonella typhi*	1(1.8)	1(1.9)	
* Campylobacter species*	0(0)	1(1.9)	

*IQR* interquartile range, *CDS* clinical dehydration scale

**Fig. 1:**
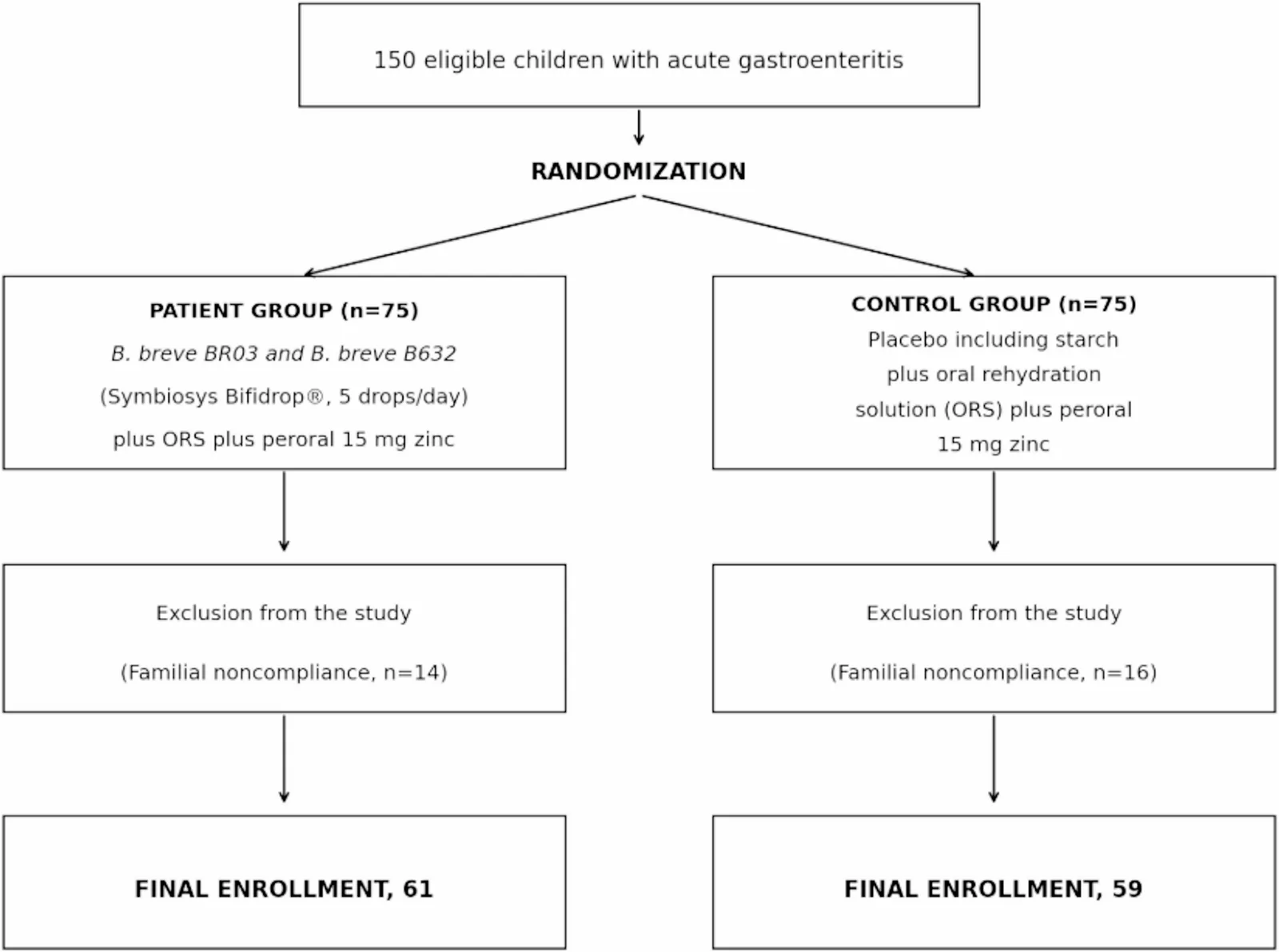
Study group flow chart

A total of 150 qualified children with the manifestation of acute gastroenteritis were enrolled and divided into two equal groups (75). Bifidobacterium breve BR03 and B. breve B632 ( Symbiosys Bifidrop(r), 5 drops/day ) were additionally given to the patient group on top of oral rehydration solution (ORS) and peroral zinc (15 mg/day) whereas the control group was given the starch-based placebo in conjunction with the ORS and the same lower dose of zinc (15 mg/day). After randomization, 14 children were discontinued within the patient group and 16 within the control group as a consequence of noncompliance within the families leading to the eventual final analysed groups of 61 and 59 participants, respectively, as shown in Fig. 1.

**Fig. 2:**
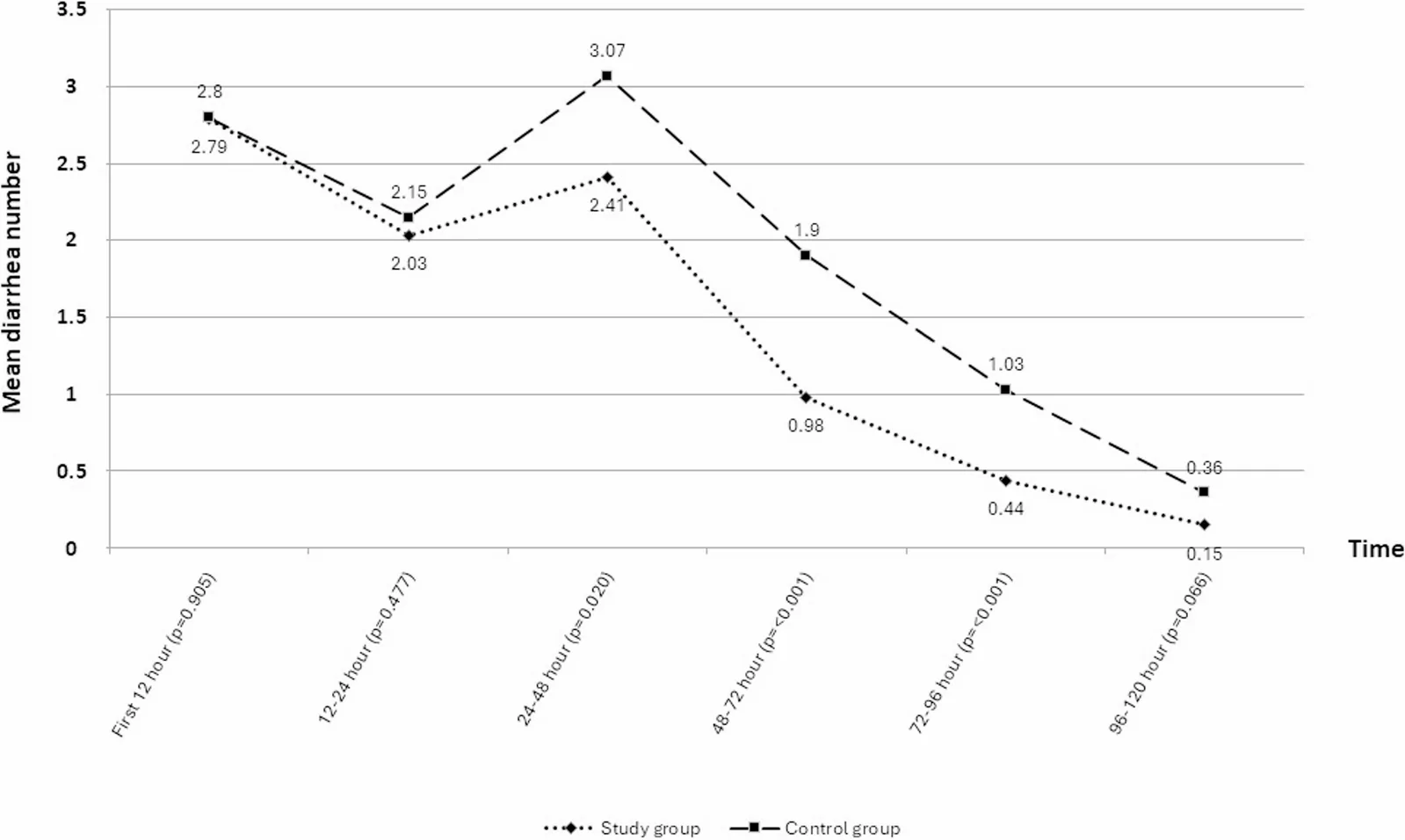
Mean diarrhea patients

Figure 2 shows that the mean stool frequency at 24–48 h was significantly lower in the study group (2.41 ± 1.99) compared to the control group (3.07 ± 1.71), with a similar trend observed at 48–72 h (0.98 ± 1.31 vs. 1.90 ± 1.58) and 72–96 h (0.44 ± 1.18 vs. 1.03 ± 1.35), where the study group exhibited consistently lower stool frequencies. These differences were statistically significant, with p-values of 0.020, *p* < 0.001, and *p* < 0.001, respectively, indicating a clear reduction in diarrhea episodes in the study group over time. These findings highlight the potential efficacy of the intervention in reducing stool frequency in children with acute diarrhea.

Table 2 shows that the proportions of children with diarrhea at 24–48 h, 48–72 h, and 72–96 h were significantly lower in the study group compared to the control group. At 24–48 h, 83.6% of children in the study group had diarrhea, compared to 94.9% in the control group, with a risk ratio (RR) of 3.660 (95% CI: 0.954–14.046; *p* = 0.046). The difference was more pronounced at 48–72 h, where 44.3% of children in the study group experienced diarrhea, compared to 78.0% in the control group (RR: 4.456, 95% CI: 2.009–9.881; *p* < 0.001). At 72–96 h, only 16.4% of children in the study group had diarrhea, in contrast to 50.8% in the control group (RR: 5.276, 95% CI: 2.258–12.325; *p* < 0.001). Although the proportion of children with diarrhea at 96–120 h was lower in the study group (8.2%) compared to the control group (20.3%), the difference did not reach statistical significance (RR: 2.860, 95% CI: 0.940–8.703; *p* = 0.057). No side effects related to the probiotic treatment were observed in any of the patients.

**Table 2:** Comparison of the ratio of children with diarrhea between study and control groups by time

	Study group (n=61)	Control group (n=59)	RR(CI 95%)	p
0-12 hr	60(98.4)	59(100.0)	-	1.000
12-24 hr	59(96.7)	55(93.2)	0.466(0.082-2.647)	0.435
24-48 hr	51(83.6)	56(94.9)	3.660(0.954-14.046)	0.046
48-72 hr	27(44.3)	46(78.0)	4.456(2.009-9.881)	<0.001
72-96 hr	10(16.4)	30(50.8)	5.276(2.258-12.325)	<0.001
96-120 hr	5(8.2)	12(20.3)	2.860(0.940-8.703)	0.057

*RR* risk ratio, *CI *confidence interval, *hr* hour

## Discussion

Probiotics are microorganisms that confer health benefits when consumed in adequate amounts, including preventing or ameliorating specific diseases such as diarrhea. Among the various probiotic strains, *Bifidobacterium breve* (B. breve) has gained attention for its therapeutic potential in addressing multiple clinical conditions, especially in pediatric populations [8]. Despite the promising benefits, research specifically investigating the effects of *B. breve* in the context of acute infectious diarrhea remains limited. This study sought to fill this gap by examining the clinical outcomes of combining *B. breve* BR03 and *B. breve* B632 in children with acute infectious diarrhea. The results revealed that the combination of *B. breve* strains led to significant improvements in the clinical outcomes of the children, particularly in the reduction of stool frequency. Mean stool counts at 24–48 h, 48–72 h, and 72–96 h were significantly lower in the intervention group compared to the control group, suggesting a rapid amelioration of diarrhea. These findings align with those reported in other studies, where probiotics have similarly been shown to reduce the severity and duration of diarrhea in various populations [9, 10]. Additionally, the proportion of children with diarrhea at 24–48 h, 48–72 h, and 72–96 h was significantly lower in the study group, further supporting the beneficial effects of *B. breve*.

The mechanisms by which probiotics benefit gut health and alleviate diarrhea are multifaceted and remain an area of active investigation. One proposed mechanism involves the ability of probiotics to maintain a healthy gut microbiota, which in turn supports the overall microbial homeostasis of the gastrointestinal tract, reducing the incidence and severity of diarrhea. A key factor in this process is the low-pH environment generated by probiotics within the gut, inhibiting harmful pathogens’ growth and proliferation [11]. Furthermore, probiotics can enhance immune responses by stimulating various immune components, thus preventing pathogen invasion. Specifically, probiotics have been shown to stimulate goblet cells and epithelial cells of the intestinal mucosa to secrete important antimicrobial substances such as mucin, cathelicidins, and defensins, all of which play a critical role in defending the gut against pathogenic microorganisms [12]. Another important mechanism involves strengthening tight junctions between intestinal epithelial cells, which further protects against the invasion of pathogenic bacteria [13].

Among the many species of probiotics, *Bifidobacteria* are particularly abundant in the neonatal gut, where they play a crucial role in the digestion of human milk oligosaccharides. Interventions aimed at increasing the abundance of *B. breve* within the gut microbiome have been associated with improved antimicrobial peptide production, which in turn contributes to a reduction in the duration of diarrhea [14]. Furthermore, symbiotic combinations containing *B. breve* are effective in animal models, where they have demonstrated protective effects against dysentery and infections caused by pathogens such as rotavirus and *Shiga* toxin-producing *Escherichia coli* O157:H7 [15–17]. In our study, however, detecting an etiological agent was limited to only approximately one-fifth of the study population. As a result, we could not further explore whether the combination of *B. breve* strains offered any distinct advantage in terms of pathogen-specific benefits.

Beyond acute gastroenteritis, *B. breve* has demonstrated therapeutic benefits in other areas, which has been shown to reduce inflammatory markers associated with cardiac hypertrophy, however, the current research primarily focused on its role in acute infectious diarrhoea.

Despite the promising therapeutic potential of *B. breve*, it is important to recognize that, although no probiotic-related side effects were observed in our study, probiotics can occasionally lead to complications, particularly in vulnerable populations such as young children and immunocompromised individuals. In rare cases, the use of probiotics has been associated with serious adverse events, including bacteremia, fungemia, sepsis, and multi-organ failure, especially when used in individuals with underlying health conditions [18, 19]. Specifically, *B. breve* has been implicated in bacteremia, with one study citing an incidence of 2% among neonates [20]. Predisposing factors for these adverse events may include intestinal mucosal damage or ileus, which can create a pathway for bacteria to enter the bloodstream. Furthermore, *B. breve* has also been linked to other severe complications, such as necrotizing fasciitis and bacteremia [21]. These risks highlight the need for careful monitoring and appropriate screening when administering probiotics to sensitive patient populations, particularly those with compromised immune systems or underlying gastrointestinal conditions.

Our findings align with previous research demonstrating the efficacy of *B. breve* in reducing the severity and duration of diarrhea in children. A randomized controlled trial by Bozzi et al. reported that supplementation with *B. breve* significantly reduced the frequency and duration of diarrhea in pediatric patients with acute gastroenteritis [22]. Similarly, a meta-analysis by Dargenio et al. concluded that probiotics, including *B. breve*, contributed to faster recovery from infectious diarrhea by modulating gut microbiota and enhancing mucosal immunity [23].

A key strength of our study is its randomized controlled design, which minimizes selection bias and enhances the validity of our findings. Additionally, using a well-characterized *B. breve* combination and standardized clinical assessment criteria strengthens the reliability of our results. However, our study has some limitations. The relatively small sample size may limit the generalizability of our findings to broader pediatric populations. Furthermore, the short follow-up period precludes an assessment of long-term probiotic effects. Another limitation is the inability to differentiate strain-specific effects, as multiple probiotic strains were used. Lastly, while an etiological agent was identified in only a fraction of cases, limiting pathogen-specific conclusions, future studies incorporating comprehensive microbiological analyses could provide deeper insights.

Future research should focus on large-scale, multicenter trials with extended follow-up periods to evaluate the long-term effects of *B. breve* on pediatric gastrointestinal health. Additionally, studies comparing different probiotic strains and dosages could help optimize treatment regimens for acute gastroenteritis. Investigating the underlying mechanisms through microbiome sequencing and metabolomic profiling would further elucidate the pathways through which *B. breve* exerts its therapeutic effects. Finally, given the emerging role of probiotics in metabolic disorders, further exploration of their potential in preventing gut dysbiosis-related conditions, such as obesity and irritable bowel syndrome, is warranted.

## Conclusion

This study provides compelling evidence that combining *Bifidobacterium breve BR03 and B. breve B632* significantly reduces the severity and duration of acute infectious diarrhea in pediatric patients without causing adverse effects. The observed reductions in stool frequency and diarrhea persistence underscore the therapeutic potential of this probiotic formulation in managing gastrointestinal infections. Our findings are consistent with previous research demonstrating the beneficial effects of *B. breve* in modulating gut microbiota, enhancing mucosal immunity, and maintaining intestinal homeostasis. While the results highlight the efficacy and safety of this probiotic intervention, further large-scale, multicenter studies with extended follow-up periods are necessary to validate these findings, elucidate strain-specific mechanisms, and optimize probiotic-based therapeutic strategies for pediatric diarrheal diseases.

## Data Availability

No datasets were generated or analysed during the current study.

## References

[1] Diarrhoeal disease [Internet] (2024) Available from: https://www.who.int/news-room/fact-sheets/detail/diarrhoeal-disease#:~:text=Key%20facts,of%20all%20diarrhoea%2Dassociated%20deaths

[2] Chu C, Rotondo-Trivette S, Michail S (2020) Chronic diarrhea. Curr Probl Pediatr Adolesc Health Care 50(8):100841 32863166 10.1016/j.cppeds.2020.100841

[3] Long CX, He L, Guo YF, Liu YW, Xiao NQ, Tan ZJ (2017) Diversity of bacterial lactase genes in intestinal contents of mice with antibiotics-induced diarrhea. World J Gastroenterol 23(42):7584–7593 29204058 10.3748/wjg.v23.i42.7584 PMC5698251

[4] Iancu MA, Profir M, Rosu OA, Ionescu RF, Cretoiu SM, Gaspar BS (2023) Revisiting the intestinal microbiome and its role in diarrhea and constipation. Microorganisms 11(9):2177 10.3390/microorganisms11092177 PMC10538221 37764021

[5] Valitutti F, Mennini M, Monacelli G, Fagiolari G, Piccirillo M, Di Nardo G et al (2025) Intestinal permeability, food antigens and the microbiome: a multifaceted perspective. Front Allergy 5:1505834 39850945 10.3389/falgy.2024.1505834 PMC11754301

[6] Szajewska H, Guarino A, Hojsak I, Indrio F, Kolacek S, Orel R et al (2020) Use of probiotics for the management of acute gastroenteritis in children: an update. J Pediatr Gastroenterol Nutr 71(2):261–269 32349041 10.1097/MPG.0000000000002751

[7] Aloisio I, Prodam F, Giglione E, Bozzi Cionci N, Solito A, Bellone S et al (2018) Three-month feeding integration with Bifidobacterium strains prevents gastrointestinal symptoms in healthy newborns. Front Nutr 5:39 29888226 10.3389/fnut.2018.00039 PMC5980983

[8] Bozzi Cionci N, Baffoni L, Gaggia F, Di Gioia D (2018) Therapeutic microbiology: the role of Bifidobacterium breve as food supplement for the prevention/treatment of paediatric diseases. Nutrients 10(11):1723 10.3390/nu10111723 PMC6265827 30423810

[9] Dinleyici EC, Dalgic N, Guven S, Metin O, Yasa O, Kurugol Z et al (2015) Lactobacillus reuteri DSM 17938 shortens acute infectious diarrhea in a pediatric outpatient setting. J Pediatr (Rio J) 91(4):392–396 25986615 10.1016/j.jped.2014.10.009

[10] Mourey F, Sureja V, Kheni D, Shah P, Parikh D, Upadhyay U et al (2020) A multicenter, randomized, double-blind, placebo-controlled trial of saccharomyces boulardii in infants and children with acute diarrhea. Pediatr Infect Dis J 39(11):e347–e51 32796401 10.1097/INF.0000000000002849 PMC7556239

[11] Peters VBM, van de Steeg E, van Bilsen J, Meijerink M (2019) Mechanisms and immunomodulatory properties of pre- and probiotics. Benef Microbes 10(3):225–236 30827150 10.3920/BM2018.0066

[12] do Carmo MS, Santos CID, Araujo MC, Giron JA, Fernandes ES, Monteiro-Neto V (2018) Probiotics, mechanisms of action, and clinical perspectives for diarrhea management in children. Food Funct 9(10):5074–5095 30183037 10.1039/c8fo00376a

[13] Zeng Q, He X, Puthiyakunnon S, Xiao H, Gong Z, Boddu S et al (2017) Probiotic mixture golden bifido prevents neonatal escherichia coli k1 translocation via enhancing intestinal defense. Front Microbiol 8:1798 28979247 10.3389/fmicb.2017.01798 PMC5611410

[14] ChenK, Jin S, Ma Y, Cai L, Xu P, Nie Y et al (2024) Adjudicative efficacy of Bifidobacterium animalis subsp. lactis BLa80 in treating acute diarrhea in children: a randomized, double-blinded, placebo-controlled study. Eur J Clin Nutr 501-508. https://www.nature.com/articles/s41430-024-01428-6.pdf 10.1038/s41430-024-01428-6 PMC11182741 38467857

[15] Rigo-Adrover MDM, van Limpt K, Knipping K, Garssen J, Knol J, Costabile A et al (2018) Preventive effect of a synbiotic combination of galacto- and fructooligosaccharides mixture with Bifidobacterium breve M-16V in a model of multiple rotavirus infections. Front Immunol 9:1318 29942312 10.3389/fimmu.2018.01318 PMC6004411

[16] Asahara T, Shimizu K, Nomoto K, Hamabata T, Ozawa A, Takeda Y (2004) Probiotic bifidobacteria protect mice from lethal infection with Shiga toxin-producing Escherichia coli O157:H7. Infect Immun 72(4):2240–2247 15039348 10.1128/IAI.72.4.2240-2247.2004 PMC375161

[17] Sharif A, Kashani HH, Nasri E, Soleimani Z, Sharif MR (2017) The role of probiotics in the treatment of dysentery: a randomized double-blind clinical trial. Probiotics Antimicrob Proteins 9(4):380–385 28321826 10.1007/s12602-017-9271-0

[18] Lolis N, Veldekis D, Moraitou H, Kanavaki S, Velegraki A, Triandafyllidis C et al (2008) Saccharomyces boulardii fungaemia in an intensive care unit patient treated with caspofungin. Crit Care 12(2):414 18423057 10.1186/cc6843 PMC2447580

[19] Sulik-Tyszka B, Snarski E, Niedzwiedzka M, Augustyniak M, Myhre TN, Kacprzyk A et al (2018) Experience with saccharomyces boulardii probiotic in oncohaematological patients. Probiotics Antimicrob Proteins 10(2):350–355 28948565 10.1007/s12602-017-9332-4 PMC5973998

[20] Sakurai Y, Watanabe T, Miura Y, Uchida T, Suda N, Yoshida M et al (2022) Clinical and bacteriologic characteristics of six cases of Bifidobacterium breve bacteremia due to probiotic administration in the neonatal intensive care unit. Pediatr Infect Dis J 41(1):62–65 34889871 10.1097/INF.0000000000003232

[21] Wakabayashi Y, Nakayama S, Yamamoto A, Yoshino Y, Ishigaki S, Furukawa T et al (2022) First case of necrotizing fasciitis and bacteremia caused by Bifidobacteriumbreve. Anaerobe 76:102613 35863723 10.1016/j.anaerobe.2022.102613

[22] Bozzi Cionci N, Baffoni L, Gaggìa F, Di Gioia D (2018) Therapeutic microbiology: the role of Bifidobacterium breve as food supplement for the prevention/treatment of paediatric diseases. Nutrients 10(11):1723 30423810 10.3390/nu10111723 PMC6265827

[23] Dargenio VN, Cristofori F, Brindicci VF, Schettini F, Dargenio C, Castellaneta SP et al (2024) Impact of Bifidobacterium longum Subspecies infantis on Pediatric Gut Health and Nutrition: Current Evidence and Future Directions. Nutrients 16(20):3510 39458503 10.3390/nu16203510 PMC11510697

